# Species versus within-species niches: a multi-modelling approach to assess range size of a spring-dwelling amphibian

**DOI:** 10.1038/s41598-020-79783-0

**Published:** 2021-01-12

**Authors:** Forough Goudarzi, Mahmoud-Reza Hemami, Mansoureh Malekian, Sima Fakheran, Fernando Martínez-Freiría

**Affiliations:** 1 Department of Natural Resources, Isfahan University of Technology, 84156-83111 Isfahan, Iran; 2 CIBIO/InBIO, Centro de Investigação em Biodiversidade e Recursos Genéticos da Universidade Do Porto, Instituto de Ciências Agrárias de Vairão R. Padre Armando Quintas, 4485-661 Vairão, Portugal

**Keywords:** Conservation biology, Ecological modelling, Biodiversity

## Abstract

Species Distribution Models (SDMs) can be used to estimate potential geographic ranges and derive indices to assess species conservation status. However, habitat-specialist species require fine-scale range estimates that reflect resource dependency. Furthermore, local adaptation of intraspecific lineages to distinct environmental conditions across ranges have frequently been neglected in SDMs. Here, we propose a multi-stage SDM approach to estimate the distributional range and potential area of occupancy (pAOO) of *Neurergus kaiseri,* a spring-dwelling amphibian with two climatically-divergent evolutionary lineages. We integrate both broad-scale climatic variables and fine-resolution environmental data to predict the species distribution while examining the performance of lineage-level versus species-level modelling on the estimated pAOO. Predictions of habitat suitability at the landscape scale differed considerably between evolutionary level models. At the landscape scale, spatial predictions derived from lineage-level models showed low overlap and recognised a larger amount of suitable habitats than species-level model. The variable dependency of lineages was different at the landscape scale, but similar at the local scale. Our results highlight the importance of considering fine-scale resolution approaches, as well as intraspecific genetic structure of taxa to estimate pAOO. The flexible procedure presented here can be used as a guideline for estimating pAOO of other similar species.

## Introduction

Species distribution models (SDMs) are a widely used tool to predict species occurrence and infer their environmental requirements^1^. Although species is the most frequently used evolutionary level to conduct SDMs, recent studies have suggested that ecological niches of intraspecific evolutionary units may significantly differ, leading to distinct responses of these units to environmental variation across their ranges^2,3^. Accounting for local adaptations of intraspecific lineages is therefore, important when predicting potential distribution of species, particularly when ‘phylogenetic niche conservatism’ (sensu Harvey and Pagel^4^) is not assumed (e.g. Banerjee et al.^5^ and Martínez‐Freiría et al.^6^).

Conservation decisions such as species’ redlisting require a deep understanding of the species distribution and abundance^7^. To assess the conservation status of species, the International Union for Conservation of Nature (IUCN) uses a number of criteria including the reduction in population size and/or geographic range over a determined time. The area of occupancy (AOO) is an index of the size of species’ geographic range which is frequently used in IUCN assessments and shows the rarity of a species through its distribution range^8^. Classifying taxa correctly into threat categories highly depends on the developed method to quantify species distribution and approximate AOO^9^. For habitat-specialist taxa, as well as small, cryptic species which require survey efforts at the fine spatial resolution, AOO would get a reliable index if it would correctly reflect the area of these specific sites^10^. SDMs are potentially applicable in estimating range measures such as AOO if appropriate spatial resolution considering resource dependency and the relevant variables affecting species occurrence are correctly selected^8^. Growing studies have indicated that using model-based approaches reduces uncertainties associated with the estimation of range size^6,11,12^.

Amphibians are ectothermic habitat-specialist vertebrates with limited dispersal ability, in which local scale environmental variables are main factors related to their occurrence^13^. A striking case occurs with spring-dweller amphibians, which are highly dependent on springs and the nearby ponds roughly all over the year. Predicting the occurrence of these species is very problematic at large scales because the presence of small water bodies such as springs is not captured in the available environmental predictors (e.g. Worldclim variables^14^), making it necessary to develop alternative approaches^15^.

**Figure 1:**
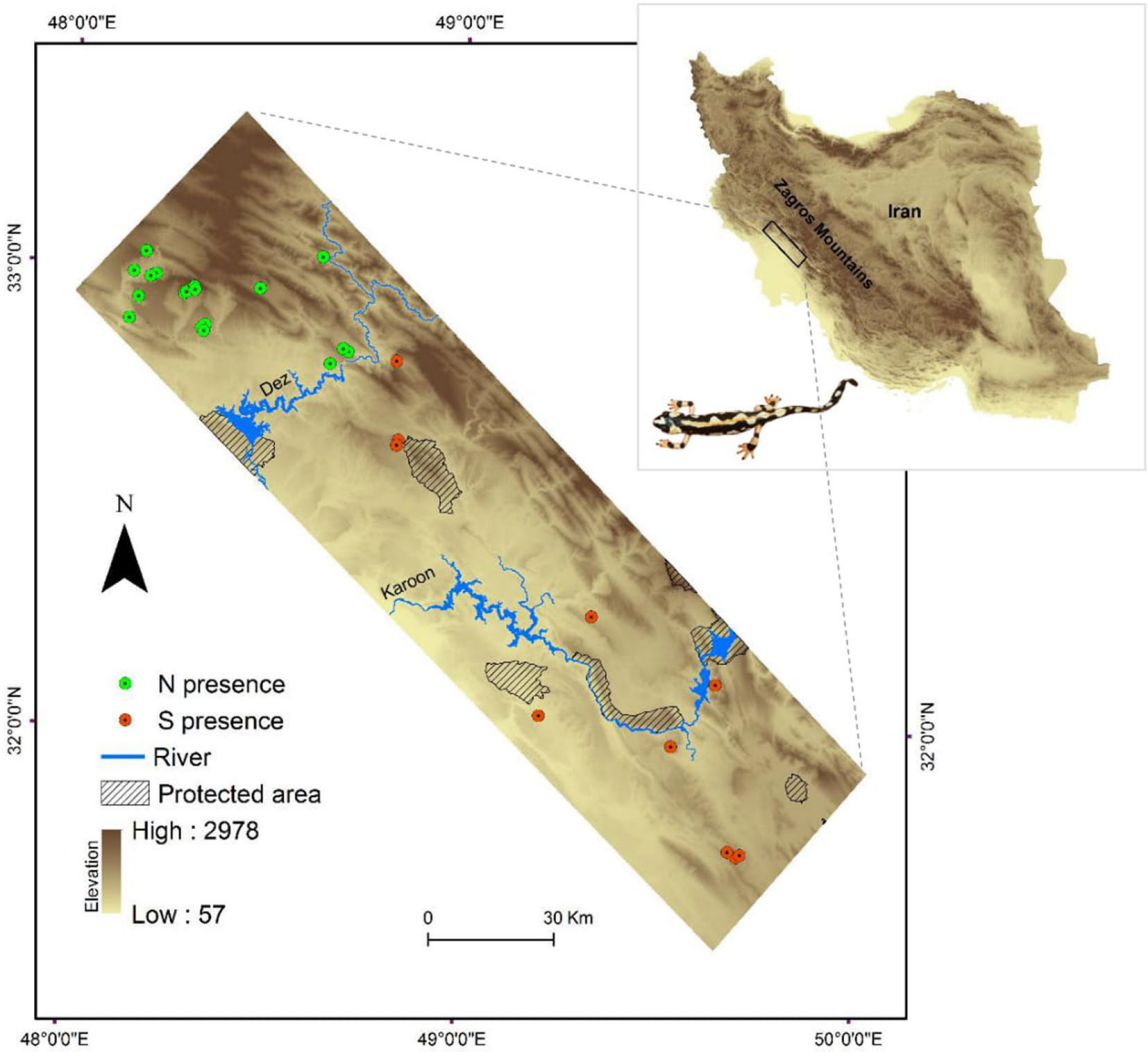
Geographic distribution of springs and ponds inhabited by the northern (N) and the southern (S) lineages of *Neurergus kaiseri* in southwestern Zagros Mountains, Iran. The maps were generated in ArcGIS 10.4 using the base free map of Digital Elevation Model from Japan Aerospace Exploration Agency (JAXA) under the Term of Use available from (https://global.jaxa.jp/policy.html). Newt is photographed by FG.

Kaiser’s newt (*Neurergus kaiseri*, Schmidt 1952) is a spring-pond dwelling amphibian^16^ distributed sporadically across southwestern Zagros Mountains of Iran^17^. Even though there is no estimate of AOO, it is currently listed as Vulnerable (VU) in the IUCN Red List due to decreasing population trend and habitat fragmentation^18^. Currently, none of the known occurrence sites of *N. kaiseri* is protected and only a small part of the predicted distribution of the species stands within the existing protected areas^19^. Two species lineages, North (N) and South (S), are recognised by both mitochondrial and nuclear DNA data, which are separated by Dez River^17^ (Fig. 1). These two lineages evolved through allopatric speciation and currently, due to no shared haplotypes in nuDNA (23,518 RAD loci), are considered as evolutionarily significant units^17,20^. Ecological niche tests have shown that these lineages occupy similar local habitats within their ancestral niche, but in different climatic conditions^17^. Environmental alterations such as climate change or habitat degradation may affect the distribution of each lineage differently^5,21,22^ and therefore, it is important to advance into conservation assessments that recognise local adaptation of populations^23^.

Here we use a multi-scale SDM approach to show the importance of including within species genetic diversity into SDMs while estimating range-based IUCN’s indices of Kaiser’s newt as a case study. We compare the estimated suitable areas for the species and its two evolutionary lineages based on different spatial scale environmental data and show the scale dependency of the results that may change conservation implications. Our specific aims are to (1) identify and compare species- and lineage-level range measures of *N. kaiseri*, and (2) integrate relevant landscape and local scale predictors in a multi-scale SDM approach. We hypothesise that: (1) the intraspecific lineage-level compared to species-level SDMs will provide a more accurate measure of *N. kaiseri*’s range size, (2) incorporating fine-scale local environmental variables improve the predictions performance of SDMs, and (3) integrating hypothesis 1 and 2 will help us in a better estimation of the species AOO.

## Results

### SDM performance

The species-level SDM exhibited good overall predictive performance at landscape and local scales (Table 1). Lineage N SDM demonstrated excellent overall predictive capacity at both landscape and local scales (Table 1). Lineage S SDM performed poorly to predict suitable habitat conditions at the landscape scale, as was evident from the low predictive capacity (AUC = 0.79, TSS = 0.27; Table 1). However, the predictive power of the S model was significantly enhanced while training under local habitat variables (Table 1).

**Table 1:** Number of records used to construct models, and performance of different evolutionary level models at the landscape and local spatial scales based on the Area Under the Curve of Receiver Operating Characteristic (AUC) and True Skill Statistic (TSS).

	N of records		Landscape scale		Local scale	
			AUC	TSS	AUC	TSS
Species-level		28	0.90	0.50	0.95	0.68
Lineage-level	N	18	0.96	0.75	0.95	0.59
	S	10	0.79	0.27	0.96	0.68

### SDM at landscape scale

The contribution of topo-climatic variables to species and lineages landscape models were different. Annual mean temperature mostly contributed to species-level modelling, while precipitation seasonality had the highest contribution to the N and S lineages models (see Supplementary Table S1 online). Univariate response curves indicated that the S lineage differently responds to both the annual mean temperature and precipitation seasonality at the landscape scale (see Supplementary Fig. S1 online).

**Figure 2:**
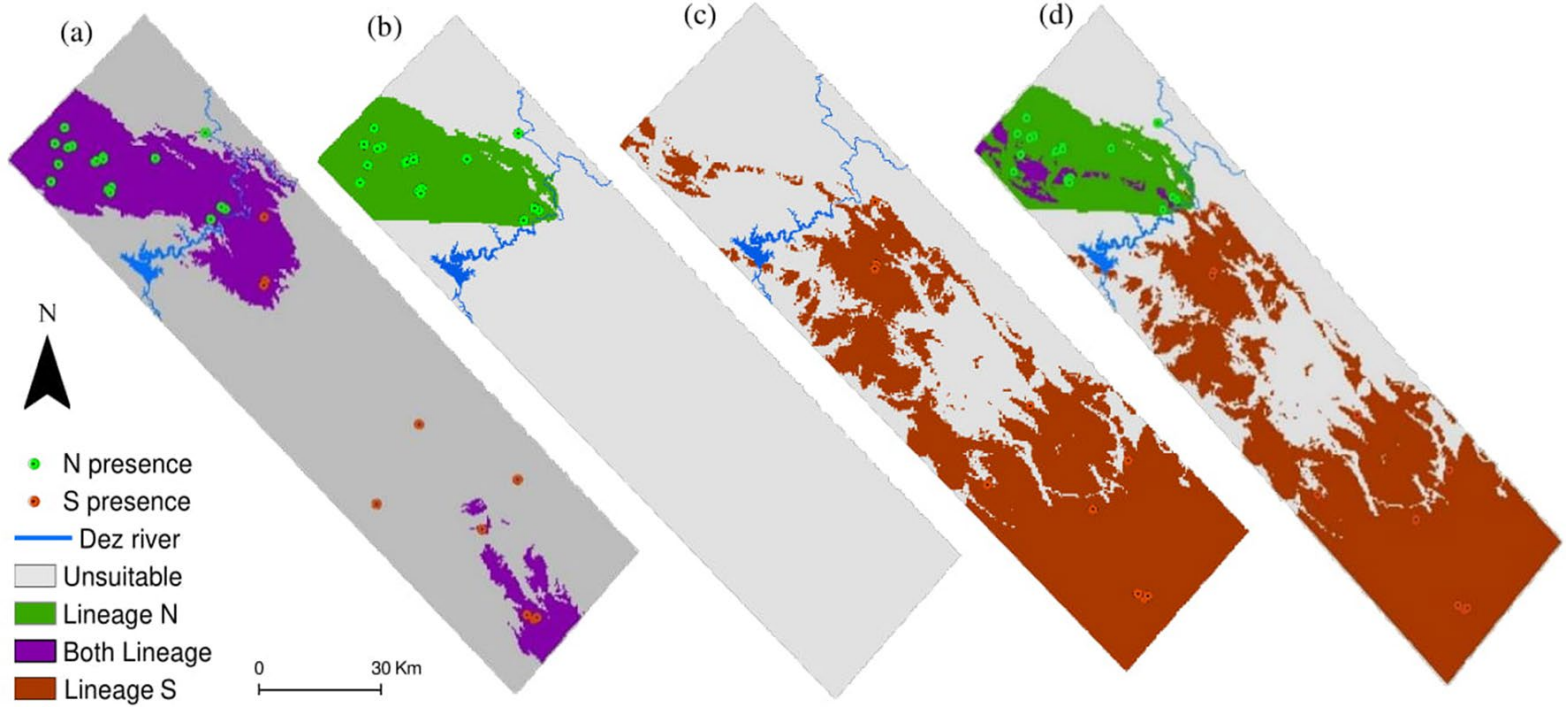
Potentially climatic suitable areas according to (**a**) Species-level binary model, (**b**) N lineage binary model, (**c**) S lineage binary model and (**d**) overlay of binary models of the two lineages (N + S binary model) for *Neurergus kaiseri* at the landscape scale. The Dez River is depicted as a reference for lineages distribution.

The predicted climatic range by the species-level model was split up as the two separate northern and southern range with no overlapped area (Fig. 2a). The climatically suitable range for N lineage was distinctively found in the north part of the study region (Fig. 2b), but the SDM predicted a wider distribution range for lineage S (Fig. 2c). Overlaying binary predictions of lineage-level models at the landscape scale revealed that the two lineages mainly occupied unique geographic ranges and had low range overlap (Fig. 2d). The probabilistic models are shown in Supplementary Fig. S2 online.

### SDM at local scale

The contribution and permutation importance of variables to species and lineages N and S local scale models were very similar. Among the six local scale variables, the contribution of forest and formation distances, and TPI, accounted for more than 77% of the three models’ (i.e. species, N and S) predictions (see Supplementary Table S2 online). The probability of occurrence of the species and both lineage N and S sharply decreased with increasing distance from forest and conglomerate formations, as well as with increasing values of TPI (see Supplementary Fig. S1 online).

**Figure 3:**
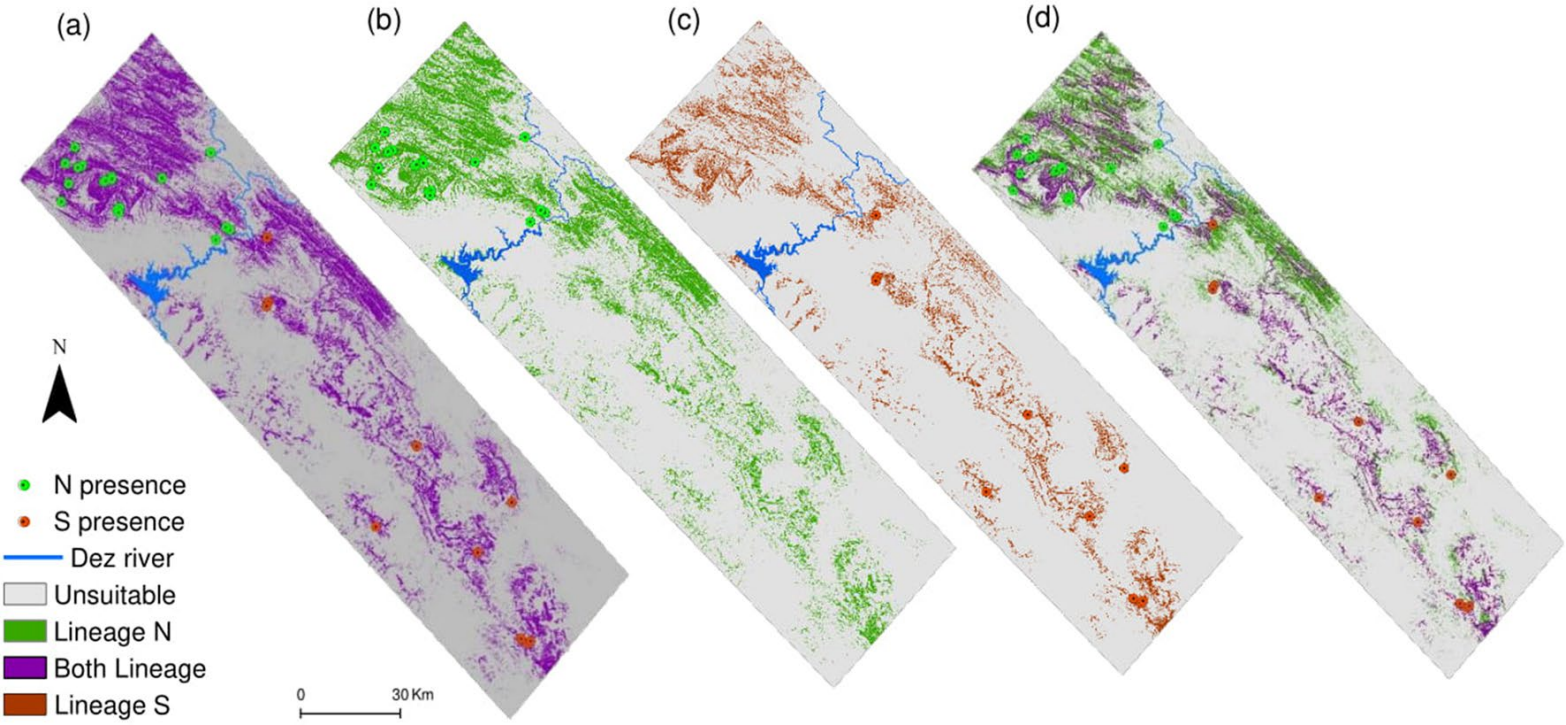
Potentially suitable areas according to (**a**) Species-level binary model, (**b**) N lineage binary model, (**c**) S lineage binary model and (**d**) overlay of binary models of the two lineages (N + S binary model) for *Neurergus kaiseri* at the local scale. The Dez River is depicted as a reference for lineages distribution.

Species-level and both lineages-level models predicted similar distribution ranges for Kaiser’s newt at the local scale (Fig. 3a). Overlaying analysis verified that lineage N and S had high geographic range overlap, with approximately no unique areas for lineage S but much unique suitable range for lineage N (Fig. 3b–d). Probabilistic models are shown in Supplementary Fig. S3 online.

### SDMs cross-prediction

The species-level model had the same successful prediction of the records at both the landscape and local scales. Predictive performance of the S model was enhanced at the local scale relative to the landscape scale (Table 2). Lineage-level models were more successful than the species-level model in predicting N and S records at the landscape scale and were the same at the local scale (Table 2). Overall, the lineage-level model had a better predictive performance. Cross-prediction tests showed that a model built with occurrence records of one lineage could not retrieve climatically suitable areas for the other lineage. At the local scale, the S model could accurately predict most of N populations’ occurrence, but the S model was not much successful (Table 2).

**Table 2:** The percentage of correct classification of each lineage’s occurrence records by species-level and lineage-level models for each of the two studied landscape and local scales, and the percentage of correct classification of each lineage’s records by another model (cross-prediction) based on minimum training presence of known occurrences.

	Landscape scale			Local scale		
	Species model	Lineage model	N	Species model	Lineage model	N
			S			S
N lineage (18 records)	94%	94%	94%	94%	94%	94%
			0%			55%
S lineage (10 records)	70%	90%	0%	90%	90%	90%
			90%			90%

### Combination of landscape and local scale models

The local scale model predicted less suitable habitat area than the landscape scale model, substantially through lineage-level modelling approach (Table 3).

**Table 3:** The number of suitable pixels (30 × 30 m) predicted by each model (N pixels), the percentage of correct classification of occurrence records by each lineage-/species-level model based on minimum training presence cut-off threshold (% CCR), and Schoener’s *D* prediction at the two evolutionary ranks.

Spatial scale		Rank	N pixels*		% CCR		Schoener’s *D*
Landscape	Species		3,141,807		85%		0.33
	Lineage	N	8,218,898	1,881,323	92%	94%	
		S		6,557,992		90%	
Local	Species		2,995,407		96%		0.86
	Lineage	N	2,810,175	2,703,309	92%	94%	
		S		1,227,324		90%	
Landscape + local	Species		1,118,135		78%		0.62
	Lineage	N	1,441,800		85%	88%	
		S				80%	

*Northern and southern lineages are shared some areas (pixels).

**Figure 4:**
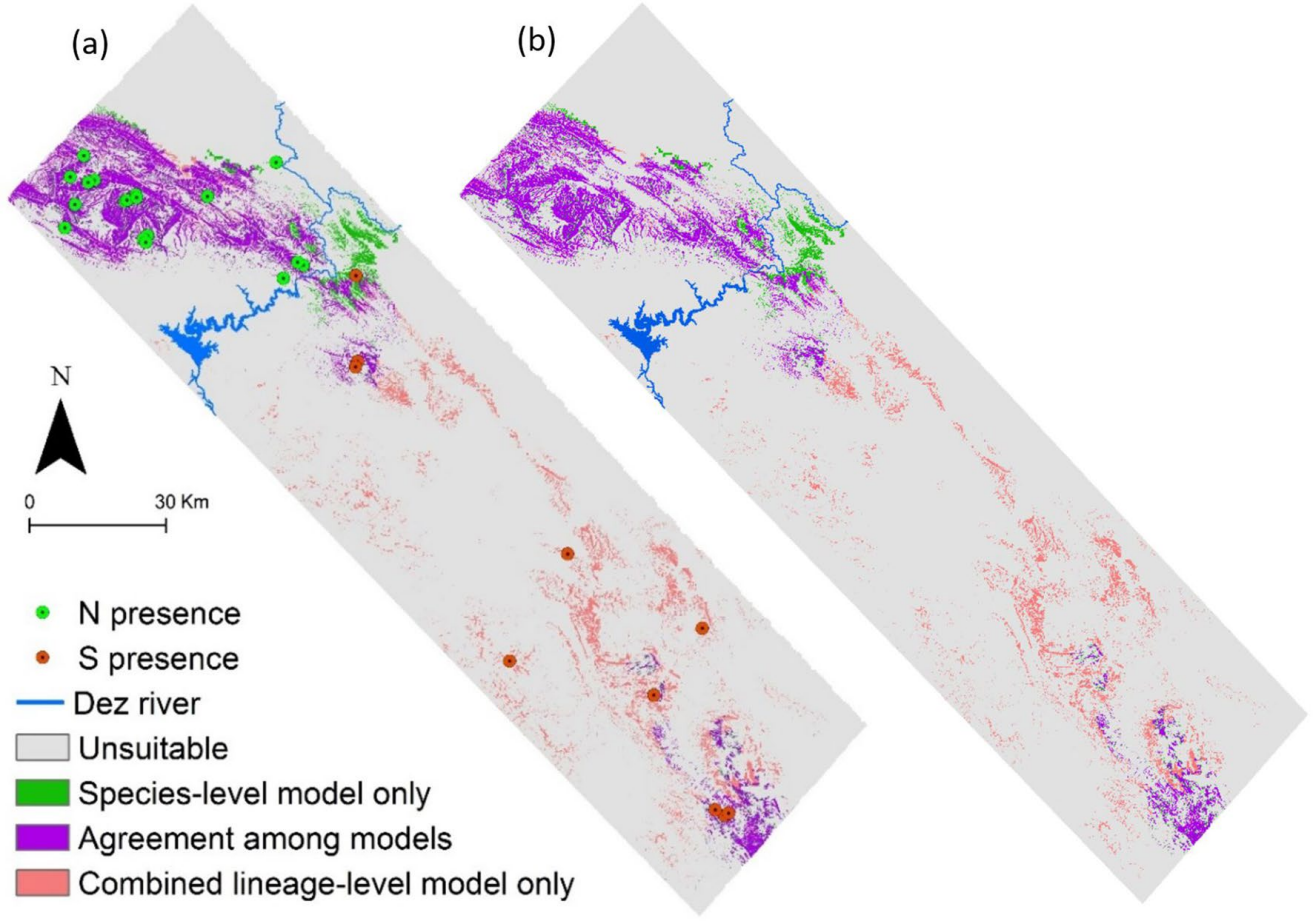
(**a**) Predicted geographic distribution of *Neurergus kaiseri* by species- and lineage-level models, derived from the combined landscape and local scale binary models. (**b**) Potential area of occupancy (pAOO) after eliminating non-spring areas at 30 m resolution. Discrepancies and agreements are depicted. The Dez River is depicted as a reference for lineages distribution.

Results of the lineage-level modelling predicted more unique, suitable habitats for Kaiser’s newt compared to the species-level model (combining all the presence points for modelling and ignoring the intraspecific distinction; Table 3, Fig. 4).

The lineage- and species-level models had low Schoener’s *D* overlap at the landscape scale (*D* = 0.33) but were very similar at the local scale (*D* = 0.86). Although, the distribution maps resulted from the two different evolutionary-level models were relatively similar (*D* = 0.62), predicted ranges were considerably different (Fig. 4).

### Potential area of occupancy (pAOO)

The suitable habitat for Kaiser’s newt accounted for 1006 and 1297 km^2^ as predicted by species-level and combined lineage-level models, respectively. Agreement of species-level and combined lineage-level models assigned approximately 805 km^2^ suitable area for the species (Fig. 4a). After overlapping with the modelled spring map, the suitable habitat for Kaiser’s newt (pAOO) was reduced by 8% and was respectively 928 km^2^ and 1186 km^2^ by species-level and combined lineage-level model (see Supplementary method and Fig. S4 online). Agreement of species-level and combined lineage-level models after eliminating non-spring areas assigned approximately 743 km^2^ suitable area for the Kaiser’s newt (Fig. 4b). Comparatively, predicted pAOO based on the IUCN standard 2 × 2 grids was estimated 3120 km^2^ by species-level model and 6364 km^2^ by combined lineage-level model. After eliminating non-spring areas, pAOO at species-level remained with the same extension, while combined lineage-level model assigned approximately 6180 km^2^ pAOO based on IUCN recommended grid size (see Supplementary Fig. S5 online).

## Discussion

We developed a multi-scale modelling approach to approximate the potential area of occupancy (pAOO) of habitat-specialist taxa with contrasting levels of intraspecific genetic and climatic niche divergence. Using the spring-dwelling amphibian *Neurergus kaiseri* as a model species, we show the importance of considering species intraspecific variability and fine-scale local environmental variables to obtain reliable predictions of distributional ranges for conservation purposes.

Our analyses recognised two climatic variables (i.e. annual mean temperature and precipitation seasonality) plus elevation as the most important parameters determining *N. kaiseri*’s distribution. Ectothermic organisms are highly dependent on environmental conditions, mainly topoclimatic, to develop their activities^8,24^. In addition, local scale variables limit species distribution within the climatic distribution. Similar to the findings of other studies on newt species^25–28^, TPI, and forest and formation distances were the most important factors limiting the species distribution range. This implies that at local scale, biophysical rather than bioclimatic variables may be important in limiting or extending species distribution range, as has also been documented by Manzoor et al.^29^. Negative TPI may allow for humidity to be retained and solar radiation to be blocked, providing a more stable local condition (suitable habitat) for Kaiser’s newt. As direct sunlight causes rapid evaporation of shallow waters in the hot and dry climate of the species range, sites incorporated enough shaded areas may be selected by the species. Canopy cover appears to supply the required shade for the northern populations, while topography provides shade for the southern populations. Several studies indicated a dependency of intraspecific lineages on distinct environmental drivers^30–32^. In our case, the S lineage could compensate for the harsh climate of the southern region by being limited to local topographic refugia^33^. Our study therefore, contributes to the growing literature which indicates that including multi-scale predictors into SDMs produces a more accurate species’ environmental niche approximation^34^, especially if within-species genetic variation is considered^21,22^.

A previous related study showed that climatic niches of the two lineages of *N. kaiseri* were not identical^17^. Consequently, we expected that the predicted landscape distribution of the species was dependent on whether to consider the species or intraspecific lineages as the unit of modelling. We found support for this expectation in the low Schoener’s *D* overlap found for topo-climatic niches of lineages, the different variable weights obtained for species- and lineage-level landscape models and the low cross-prediction capacity of lineage-level models. In contrast, at the local scale at which lineages had identical occupied niches^17^, the predicted areas by the two different evolutionary-level models were very similar. Also, both species and lineage models were affected by the same local variables, i.e. forest distance, formation distance and TPI, and the associated response curves showed a similar profile for these three factors.

We found that incorporating intraspecific genetic information firmly improves the prediction of SDMs. The combination of topo-climatic niches of both lineages provided a wider range size compared to the topo-climatic niche inferred for the species; consequently, spatial predictions differed at these two evolutionary levels. Due to unequal sample size, the species-level model was biased in favour of lineage N, which likely resulted in losing variables’ dependency and consequently reducing the suitable range of lineage S (see Pearman et al.^21^). The lineage-level models predicted the occurrence sites with higher accuracy compared to that of the species-level model and identified climatically suitable unique areas within the distribution range of both lineages (i.e. high sensitivity). A comparable pattern was recently reported by Lecocq et al.^22^, and Chardon et al.^30^. Still, our inferences are based on small sample size (n < 30), and although we used a robust algorithm capable to deal with small sample size^35^, our findings must be considered with caution^36^. Increasing studies are demonstrating the necessity of considering intraspecific lineages in SDMs, particularly those with unique evolutionary histories^6,37^, and predicting the response of lineages to anthropogenic climate change^5,21,22,38^. Our findings revealed that the two lineages of the Kaiser’s newt may act differently in response to climate change and should be considered as separate conservation management units.

In a heterogeneous landscape, habitat-specialist species occupy only a small fraction of their extent of occurrence that represents the AOO. Kaiser’s newt is limited to particular topo-climatic and habitat conditions occurring nearby springs and ponds in the South-West Zagros Mountains. Our study is the first applying a SDM to develop a multi-stage, fine-grid-based procedure for approximating the potential AOO of a spring-dwelling amphibian. As suggested by Jiménez-Alfaro et al.^11^, we used fine-spatial-scale data to reduce the uncertainty of our SDMs for estimating AOO. The use of SDM for AOO estimation has been criticised due to probable extinction events across the predicted potential suitable range of species^39^. However, we show it can be useful for rare species where detectability hampers a good knowledge of distributional range^39^, particularly when appropriate spatial resolution mirroring resource (i.e. spring/ponds) dependency is considered in model building^8^.

The IUCN recommends a standard 2 × 2 km resolution to estimate AOO, which frequently requires downscaling or upscaling distributional ranges^40^. Distinct works have pinpointed that downscaling ranges for estimating AOO may result in overestimations of this metric^11,41^, especially for freshwater taxa^25^. Here, we recommend developing taxon-specific standard resolutions to homogenously estimate AOO for conservation applications. In our study, the use of a very fine grid size (30 × 30 m) for the final model to match the area of species core breeding habitat reduces overestimations in the pAOO. Still, our pAOO estimation can be upscaled and standardised based on IUCN recommendation for determining the conservation status of a species. Considering the relatively similar habitat use pattern of spring-dwelling amphibians, the developed procedure for estimating pAOO can be used for other spring dwellers worldwide. The developed modelling process can be improved by including finer scale predictors such as microclimate data and biological covariates (e.g. larvae predators and competitors) if available.

Our results highlight the importance of incorporating fine-scale environmental variables, as well as intraspecific genetic information to estimate AOO. Incorporating this all information will provide us with a more accurate understanding of the species distribution/AOO for redlisting. Grid-based, standard approaches are known to introduce an important bias into range measures, particularly in habitat-specialist species^25^. Eventually, bias may result in misclassifications of taxa on the Red List of threatened Species^24^. Where possible, conservation planning for spring-dwelling species should be informed by output of lineage-based rather than species-level SDMs incorporated with fine-scale environmental variation.

## Methods

### Model species and study area

The Zagros Mountain range represents the southern, Asian branch of the Alpine geosynclines. Annual precipitation of over 500 mm has formed a relatively dense oak forest. Karstic carbonate aquifers, which are highly fed during the wet season, are characteristic of these mountains; where they meet in contact with nonkarstic formations or alluvium, water emerges as spring^42^.

Kaiser’s newt is endemic to the South-West Zagros Mountains of Iran^18^, patchily occurring in springs surrounded by woodland with rock outcrops. We delimited a study area of 12,635 km^2^ covering the entire identified distribution range of the species and some potentially suitable localities (Fig. 1). The climate through the study area varies from wet in the north to dry in the south, mainly due to the influence of dry-warm weather from the Arabian Peninsula in the south-west. The elevation ranges from 57 to around 3,000 m above sea level. Land use pattern includes intermixed oak woodland and cropland (mainly dry farming), urban and rural residential, and transportation development leaving a preponderance of forest cover on higher elevations. Lowland steep slopes are dominated by deciduous and evergreen shrubs with more than 30% cover and desert steppes with over 10% cover^43^.

We conducted two systematic surveys during April-June in 2015 and 2016 to locate occupied sites of rare Kaiser’s newt. The sampling effort included all known permanent breeding sites of the species reported by the Department of Environment (DOE) and previous studies^44,45^. We also recorded and verified new localities (n = 6) with the assistance of local people. To reduce observer heterogeneity bias, all occupied springs/ponds were surveyed similarly by one person through dip-netting^46^. We recorded a total of 30 sites (encompassing springs and neighbouring ponds) at GPS resolution inhabited by adults and/or larvae of Kaiser’s newt (Fig. 1). Our recorded localities represented the known range of *N. kaiseri* as of the final collection in 2017. We avoided the inclusion of eight further records available in the literature (e.g. Mobaraki et al.^45^ and Vaissi and Sharifi^19^) due to their coarse-scale resolution. The average migration distance in closely related species (e.g. crested newts) is about 400 m^47^. Hence, to ensure preserving at least 500 m distances between localities, we spatially trimmed occurrence points. The final dataset comprised of 28 occupied sites by the whole species, including 18 by the lineage N and 10 by the lineage S considering the evolutionary history of the species.

### Spatial predictors

#### Landscape scale predictors

Climate is the primary driver of species distribution by limiting individuals’ establishment and dispersal^48^. Freely available variables, such as the bioclimatic variables from Worldclim^14^ (http://www.worldclim.org/bioclim), are likely spatially biased for our study area due to the scarce number of weather stations across the region; furthermore, these variables do not match to our sampling period and are not available for lower resolutions than 30 s (~ 1 km^2^). Therefore, we derived the 19 bioclimatic variables from temperature and rainfall data recorded over 13–26 recent years at 20 synoptic stations around our study region using the ‘biovars’ function in the ‘dismo’^49^ package in R^50^. These 19 variables at ~ 500 m resolution were interpolated using the Spline algorithm that accounts for the elevation (Japan Aerospace Exploration Agency; JAXA) in ArcGIS 10.4^51^. We selected the input variables considering the physiological and ecological requirements of newts and a comprehensive literature review^52–56^. Furthermore, variables were tested for correlation and only a set of low correlated variables (Pearson’s pairwise correlations ≤ 0.75) composed of five bioclimatic (annual mean temperature, Bio1; mean temperature diurnal range, Bio2; temperature seasonality, Bio4; annual precipitation, Bio12; and precipitation seasonality, Bio15) plus elevation were retained for further analyses.

#### Local scale predictors

Incoming Solar Radiation (SR), Topographic Wetness Index (TWI) and Topographic Position Index (TPI) were extracted from 30 m resolution Digital Elevation Model (DEM) (Japan Aerospace Exploration Agency; JAXA) in ArcGIS 10.4^51^ and used as a proxy of the local climate. SR affects habitat conditions and contains the information on aspect, slope, and latitude^57^. The spatial variation of solar radiation (Wh/m^2^) is severely influenced by the topography at the landscape scale, which along with temporal (daily and annual) variation contributes to local climate variability. Average solar radiation was calculated hourly for the 1^st^ and 15th of each month from March to October (active season of the study species) using the Solar Radiation tool of the ArcGIS^51^ Spatial Analyst extension. TWI is a steady-state wetness index used for quantifying effects of topography on hydrological processes, such as surface flow and is calculated from the flow accumulation across the landscape. TPI is an index of topographic roughness and compares the elevation of each cell in a DEM to the mean elevation of a specified neighbourhood around that cell (local window). As TPI is naturally very scale-dependent, it was calculated relative to 10, 30, 50, 70, 90, 110, 130 and 150 m neighbourhoods using the Land Facet Corridor Tool^58^. The absolute value of TPI increased with increasing neighbourhood size up to 90 m, and then remained unchanged beyond that; thus, a 90 m neighbourhood TPI was considered the most representative of the terrain roughness of the study area and was used for modelling. Negative values represent grids that are lower in elevation than their surrounding (e.g. ravines), positive values represent grids that are relatively higher (e.g. ridge) and zero means a flat or low slope area. In addition, NDVI (the Normalised Difference Vegetation Index) was derived from non-cloudy images of Landsat-7 ETM (9 and 15 June 2015). We considered the influence of the terrestrial habitat surrounding breeding sites by measuring the distance from breeding sites to the edge of the forest, Forest distance. We used a worldwide 30 m resolution land cover map produced by Chen et al.^43^. According to this map, the forest in the study area includes a canopy cover of more than 30% and sparse woodlands with a cover of 10–30%.

Many ecological studies on amphibians have concluded that geological substrate is a key driver of amphibian habitat suitability^28,59^. We used the National Iranian Oil Company’s 1:100,000 geological map and considered distance to conglomerate formation across the study area as a geological predictor, Formation distance. In the study region, conglomerate substrates (i.e. Kashkan and Bakhtiari formations) have high porosity and the capability to store phreatic water^27^. This physical property provides water and hiding cavities for newts; hence, it increases the probability of their presence. Since there were no significant pairwise, correlated variables, as tested using the Band Collection Statistics tool in ArcGIS^51^, all six variables were employed in the model building.

### Multi spatial scale distribution modelling

Collecting true absence data is difficult in species with secretive behaviour such as most of the amphibian species. Lack of true absences hampers the use of presence/absence approaches, which are based on the strong assumption that species are perfectly detected. We used Maxent V.3.4.1^60^, a presence-background method for model building, which has a high predictive power even when there is only a small sample of presence points^60^, and has been widely used in spatial ecology (e.g. Martínez‐Freiría et al.^6^, Rodríguez-Rodríguez et al.^3^, Banerjee et al.^5^).

We selected a total of 11 predictive variables gathered at distinct spatial scales (i.e. landscape and local scale). Integrating all of these variables in a unique model would imply upscaling the local variables from 30 to 500 m resolution, causing the loss of fine-scale habitat information. On the other hand, considering our small sample size, incorporating many predictors introduce complexity that would affect model prediction^61^. Hence, we implemented a two-step modelling procedure dividing the selected variables into the two steps.

#### SDMs development

We used three occurrence datasets for model building: (1) all occurrences for the species (28 records), (2) occurrence of lineage N (18 records), and (3) occurrence of lineage S (ten records). Our approach followed these steps (see Supplementary Fig. S6 online): (1) Landscape and local predictors were separately employed to construct species-level and lineage-level models in Maxent (six distinct models in total); (2) Average variable contribution and permutation importance of the models, as well as response curve profiles, were considered to identify the most important variables affecting the distribution of the species and lineages at both landscape and local scales; (3) Minimum training presence logistic threshold (MTP) was used to convert the continuous predictions generated by Maxent to binary predictions due to its capacity in classifying all the localities with records of *N. kaiseri* as suitable (see Supplementary Note and Table S3 online); (4) N and S binary models were overlaid and compared to the species-level binary model to assess the predictive power of the two different evolutionary level SDMs (species‐level model versus lineage‐level models); (5) Binary landscape scale models were resampled into 30 m resolution and overlaid with local scale models at the two different evolutionary levels (i.e. species- and lineage-level). Congruent pixels depicting suitable areas at both spatial scales were selected.

We developed all Maxent models with the following settings. We ran 30 bootstrap replicates for N and S populations. A 75/25 ratio was devoted to randomly splitting the occurrence data into train/test in model building. We implemented the random seed option to guarantee utilising a separate train/test dataset in every run. Recent studies have claimed that using the default setting is not always the appropriate option (e.g. Merow et al.^62^; Radosavljevic and Anderson^63^) especially when the sample size is small^64^. In addition, the number of model parameters affects model complexity, which may cause over-fitting^65^. To control the model complexity and over-parameterisation, we calculated the *beta* multiplier and also reduced the number of included features and covariates^66^. We ran the model using different regularisation *beta-*multiplier values (0.5, 1, 2) and assessed the most parsimonious N and S models based on Akaike’s Information Criterion corrected for small sample size (AIC_c_). Accordingly, the final models were built with a *beta*-multiplier of 1. We also selected different features based on the available number of occupied sites for each model: Linear, Quadratic and Hinge for lineage N with 18 records and Linear and Quadratic for lineage S with 10 records^67^.

#### Model evaluation

For assessing the performance of the models at the landscape and local scales (hypothesis 1) in discriminating suitable/unsuitable areas, we took the advantages of two evaluators. To show the general accuracy of the models, we used the Area Under the Curve of Receiver Operating Characteristic (AUC), and to evaluate the predictions after being transformed with the selected threshold, the True Skill Statistic (TSS)^68^ was employed^6,21^. For assessing the predictive performance of different models at different evolutionary ranks (hypothesis 2), we calculated the percentage of correct classification records by each of the intraspecific lineages and binarised species SDMs based on MTP cut-off threshold. To calculate sensitivity (proportion of correctly identified presences) we employed the recorded *N. kaiseri* occurrences which used to calibrate the SDMs. We also quantified the similarity of the prediction of the two model types based on Schoener’s *D* index^69^ using ENMtools^70^. For more comparison, we also calculated the suitable area predicted by each model type.

#### Potential occupancy areas

Kaiser’s newt is a spring-breeder; a characteristic derived from its stream-breeding ancestors^16^. To find the sites potentially suitable for being occupied by Kaiser’s newt, we masked the two models (i.e. species and combined lineage-level model) by a spring map produced through a distribution modelling approach (see Supplementary Method online). This assisted to eliminate the areas other than spring sites where the species could not persist. We also estimated the AOO adapted from the IUCN Red List criteria (i.e. 2 × 2 km grid cells^40^), and ran through the R^50^ package ‘Redlistr’^71^. We specified that at least one per cent of a grid cell should be potentially occupied to be counted in estimating pAOO.

## Supplementary Information

Supplementary Information.

## Data Availability

Distribution data will be available on reasonable request.
