# Supplementary material for: Species versus within-species niches: a multi-modelling approach to assess range size of a spring-dwelling amphibian
Source: Sci Rep. 2021 Jan 12;11:597. doi: 10.1038/s41598-020-79783-0 (PMC7804274; doi:10.1038/s41598-020-79783-0)
Supplement: Supplementary file 1 — Supplementary Information. [file 41598_2020_79783_MOESM1_ESM.docx]

Species versus within-species niches: a multi-modelling approach to assess range size of a spring-dwelling amphibian

**Forough Goudarzi^1^, Mahmoud-Reza Hemami^1*^, Mansoureh Malekian^1^, Sima Fakheran^1^, Fernando Martínez-Freiría^2^**

^1^ Department of Natural Resources, Isfahan university of Technology, Isfahan, 84156-83111, Iran

2 CIBIO/InBIO, Centro de Investigação em Biodiversidade e Recursos Genéticos da Universidade do Porto, Instituto de Ciências Agrárias de Vairão R. Padre Armando Quintas, 4485-661 Vairão Portugal

*Corresponding author: M.R. Hemami (email: [mrhemami@cc.iut.ac.ir](mailto:mrhemami@cc.iut.ac.ir), Tell: +98-31-3391-1031), ORCID: 0000-0002-8321-6776

**Supplementary Table S1** Contribution and permutation importance of topo-climatic variables used to fit Maxent models of *Neurergus kaiseri* distribution at landscape scale.

| Variables | Percent Contribution | | | Permutation importance | | |
| --- | --- | --- | --- | --- | --- | --- |
|  | Species-Level | Lineage-Level | | Species-Level | Lineage-Level | |
|  |  | N | S |  | N | S |
| Elevation | 22.5 | 3.8 | 34.3 | 10 | 2.1 | 39 |
| Bio1 | 23.7 | 18.6 | 8.2 | 40.4 | 19.5 | 17.1 |
| Bio2 | 23.6 | 14.4 | 0.9 | 16.6 | 11.9 | 1 |
| Bio4 | 5.6 | 5.2 | 13.2 | 7.8 | 7 | 14.4 |
| Bio12 | 5.2 | 5 | 8.4 | 6.4 | 13.8 | 5.4 |
| Bio15 | 19.5 | 53 | 34.9 | 18.8 | 45.7 | 23.1 |

**Supplementary Table S2** Contribution and permutation importance of local scale variables used to fit Maxent models of *Neurergus kaiseri* distribution at Local scale.

| Variables | Percent Contribution | | | Permutation importance | | |
| --- | --- | --- | --- | --- | --- | --- |
|  | Species-Level | Lineage-Level | | Species-Level | Lineage-Level | |
|  |  | N | S |  | N | S |
| Forest distance | 34.1 | 43.7 | 28 | 55.1 | 29.9 | 30.2 |
| Formation distance | 21.7 | 19.2 | 31.2 | 7.4 | 30.9 | 51 |
| TPI | 24.1 | 15 | 20.8 | 24.4 | 12.3 | 9.4 |
| TWI | 12.2 | 11.6 | 6 | 4.1 | 5.3 | 0.6 |
| Solar radiation | 7.1 | 8.1 | 10.2 | 7.8 | 19.2 | 5.2 |
| NDVI | 0.8 | 2.4 | 3.8 | 1.1 | 2.4 | 3.6 |


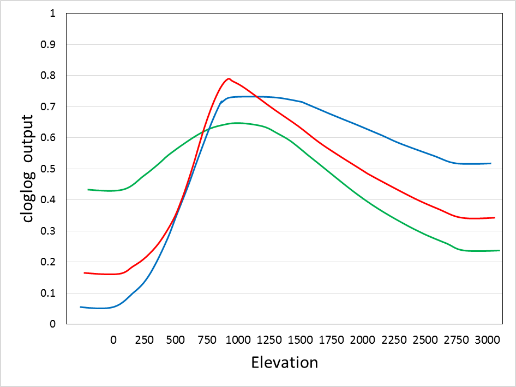

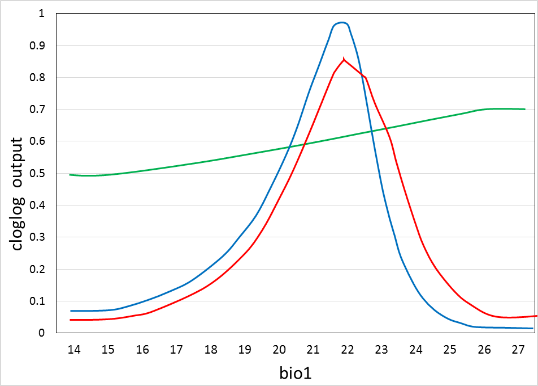

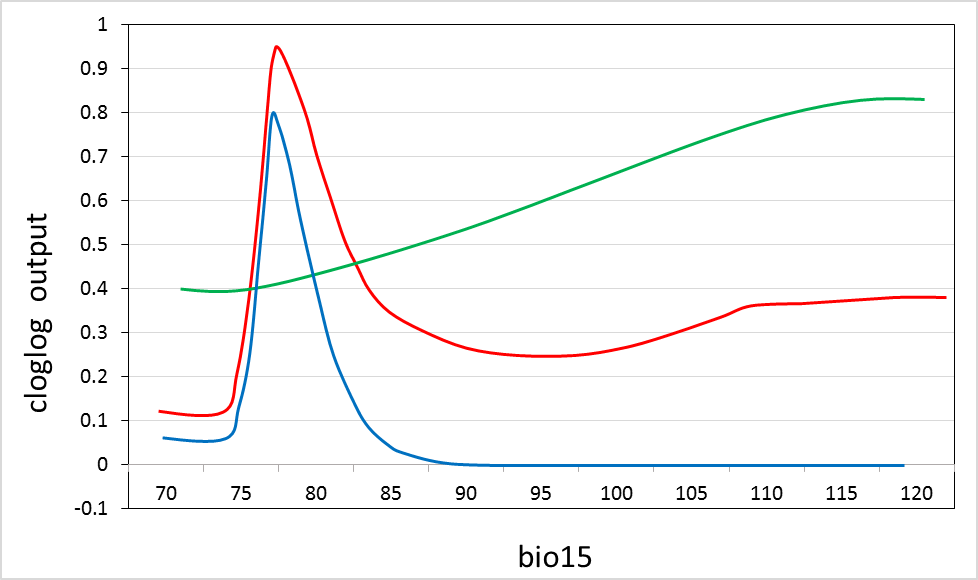

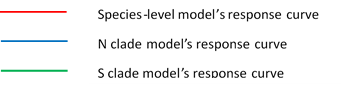


1. Response curves at landscape scale
2. Response curves at Local scale


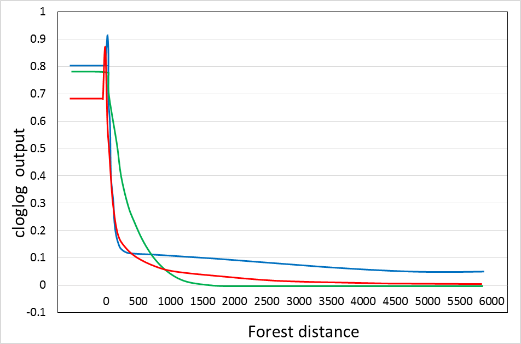

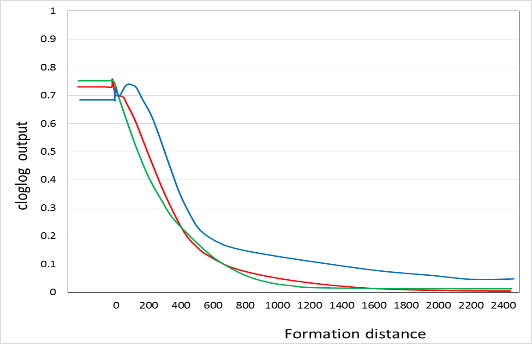

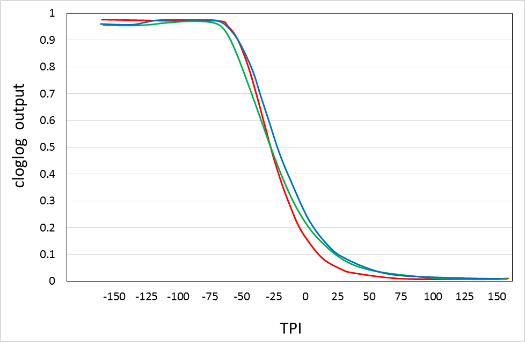


**Supplementary Figure S1** Univariate response curves for the most important common variables for each of the three distribution models (Species level, Northern lineage and Southern lineage models) of *Neurergus* *kaiseri* at (a) landscape and (b) local spatial scale.


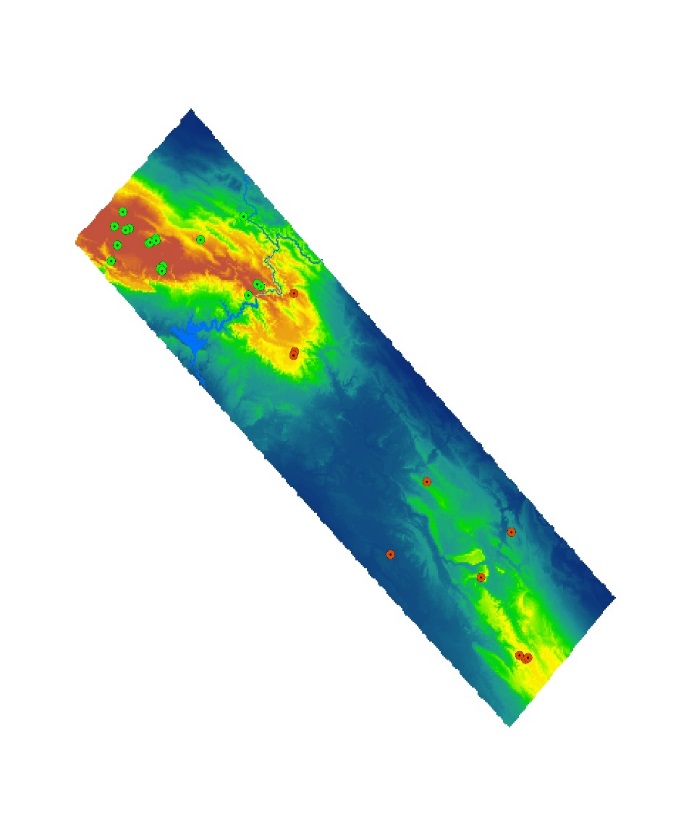

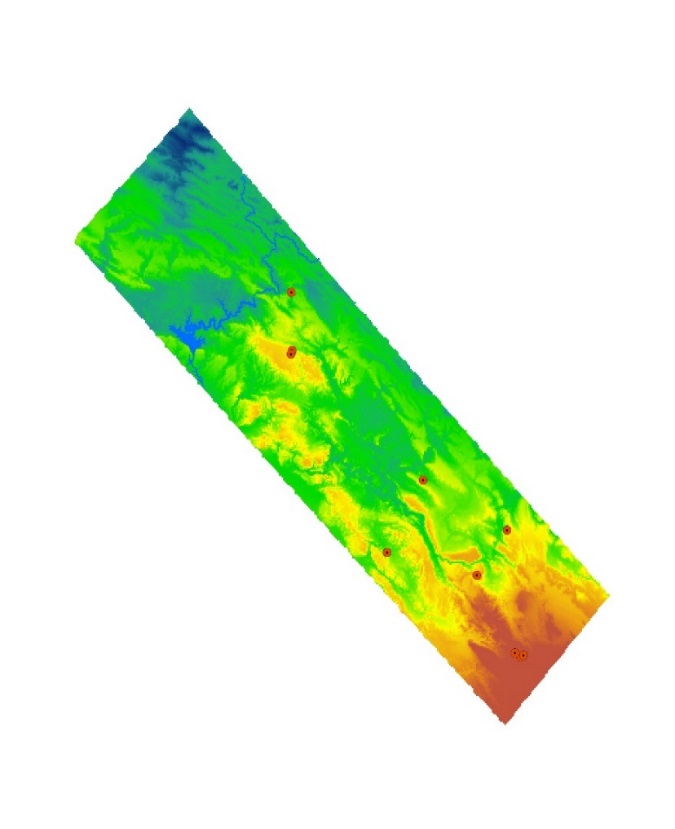

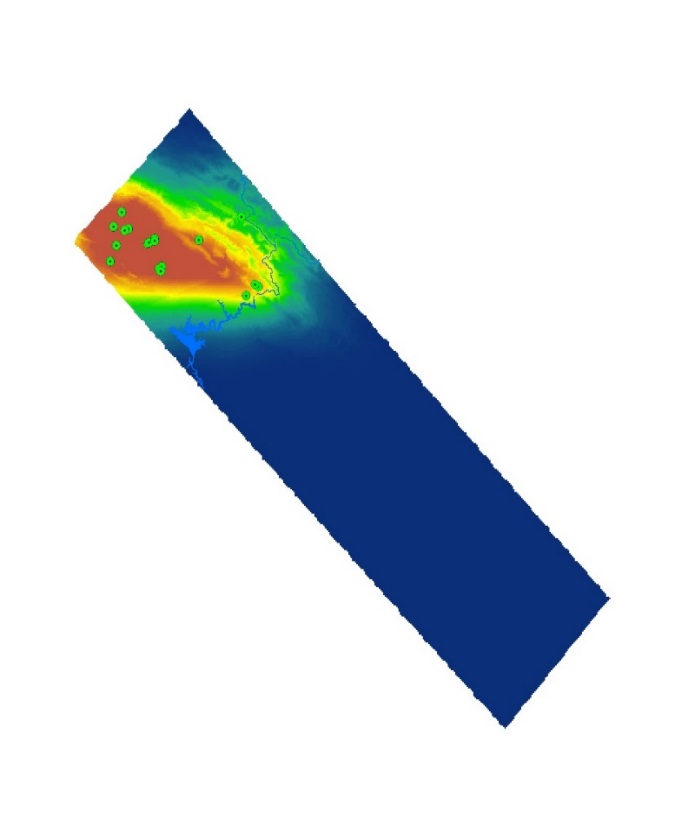


(a)

(b)

(c)

**Supplementary Figure S2** The probabilistic distribution maps according to (a) Species-level model, (b) Northern lineage model, and (c) Southern lineage model for *Neurergus* *kaiseri* at the landscape scale.


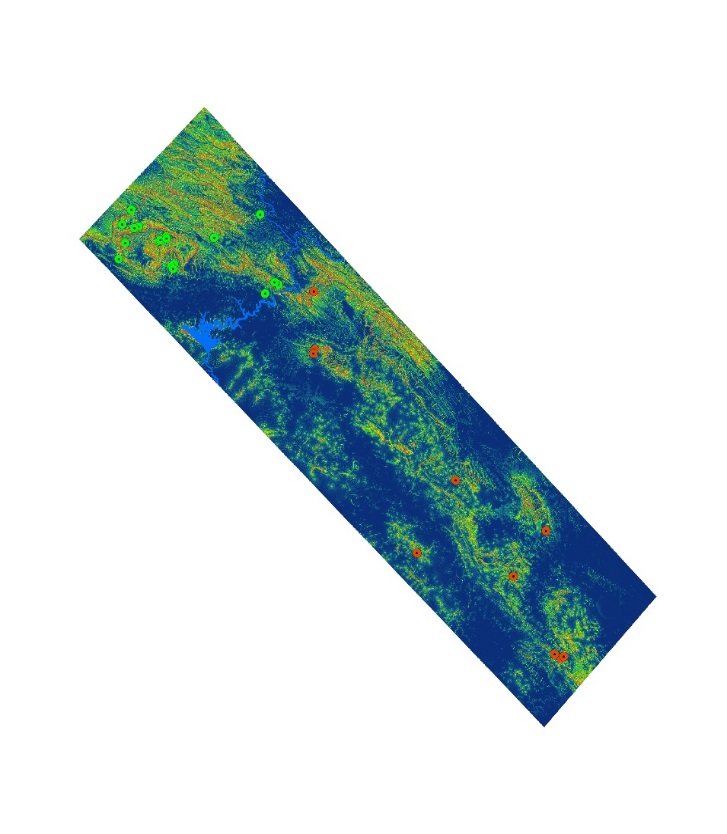

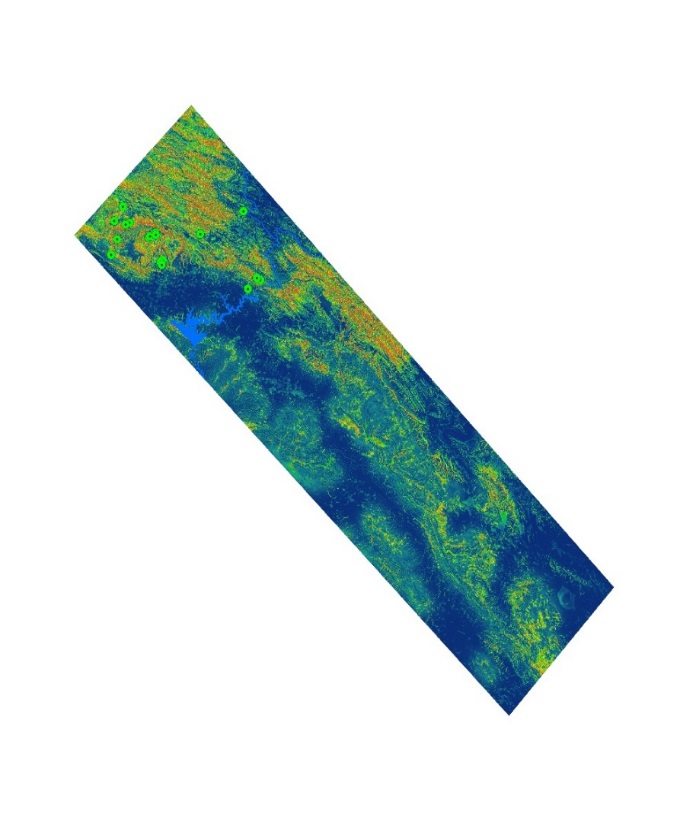

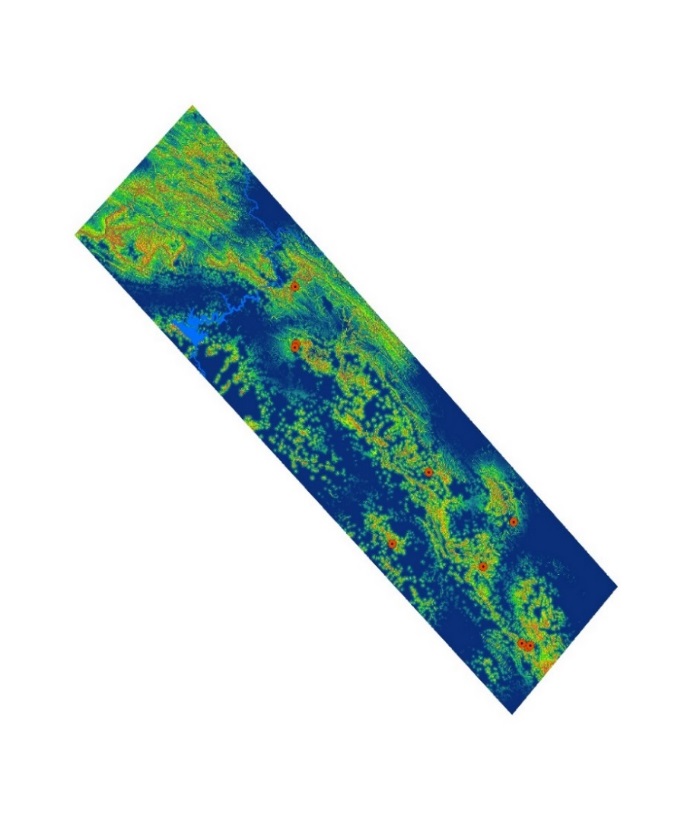


(a)

(b)

(c)

**Supplementary Figure S3** The probabilistic distribution maps according to (a) Species-level model, (b) Northern lineage model, and (c) Southern lineage model for *Neurergus* *kaiseri* at the local scale.

**Supplementary Method**

*Potential area of occupancy*

To find the sites potentially suitable for being occupied by Kaiser’s newt (pAOO), we modeled the distribution of potential spring sites across the known species range. Because of dense vegetation and/or low TPI at the location of springs, Normalized Differences Water Index (NDWI) was not reliable to identify and map springs (even when using sentinel-2 level 1C dataset with 10 m resolution; [Drusch et al.^1^](#_ENREF_1)). For this reason, in mapping springs we used TWI that is commonly investigated as an indicator for static soil moisture content [^2^](#_ENREF_2). We trained a Maxent model using the coordinates of 78 springs (28 occupied and 50 unoccupied springs by the species), and a number of environmental variables suggested in previous studies[^3^](#_ENREF_3). We included elevation, distance to the nearest fault, geology, land cover, plan curvature, distance to rivers, slope length, Stream Power Index (SPI), Topographic Wetness Index (TWI), slope, and aspect. Slope and aspect were not included in the final spring model because of their negative effect on the gain of the model. The Maxent model was fitted and evaluated based on a 10-fold cross-validation data set. For assessing the spring model accuracy (hypothesis 3), TSS and Continuous Boyce Index (CBI) were calculated. We also reclassified the output map as suitable and unsuitable areas for the presence of potential springs based on MTP threshold. Finally, we masked the species and combined lineage-level model by the produced spring map to eliminate the areas other than spring sites. To assess the accuracy of the masked maps, we manually examined the spatial position of each occupied/unoccupied spring. Subsequently, the overall accuracy, sensitivity, and specificity of the masked maps were calculated.

The average AUC for the potential spring distribution model was 0.77 (0.08 SD), the average TSS was 0.36, and the CBI was 0.95 representing an acceptable performance of the model in mapping springs across the study area.

Geology was the most influencing factor determining spring presence. After eliminating non-potential areas for emerging springs from the distribution map, the suitable area was reduced to 1186 km^2^ and 928 km^2^ based on species-level and lineage-level models, respectively, for Kaiser’s newt (Supplementary Fig. S5). The final lineage-level masked map represented the potential area of occupancy of Kaiser’s newt with an overall accuracy of 0.87, and high median sensitivity of 0.89 confirmed that the final model was powerful enough for predicting suitable breeding sites of Kaiser’s newt. The specificity of the model was 0.85 indicating that the model partly overestimates areas potentially occupied by the newt.


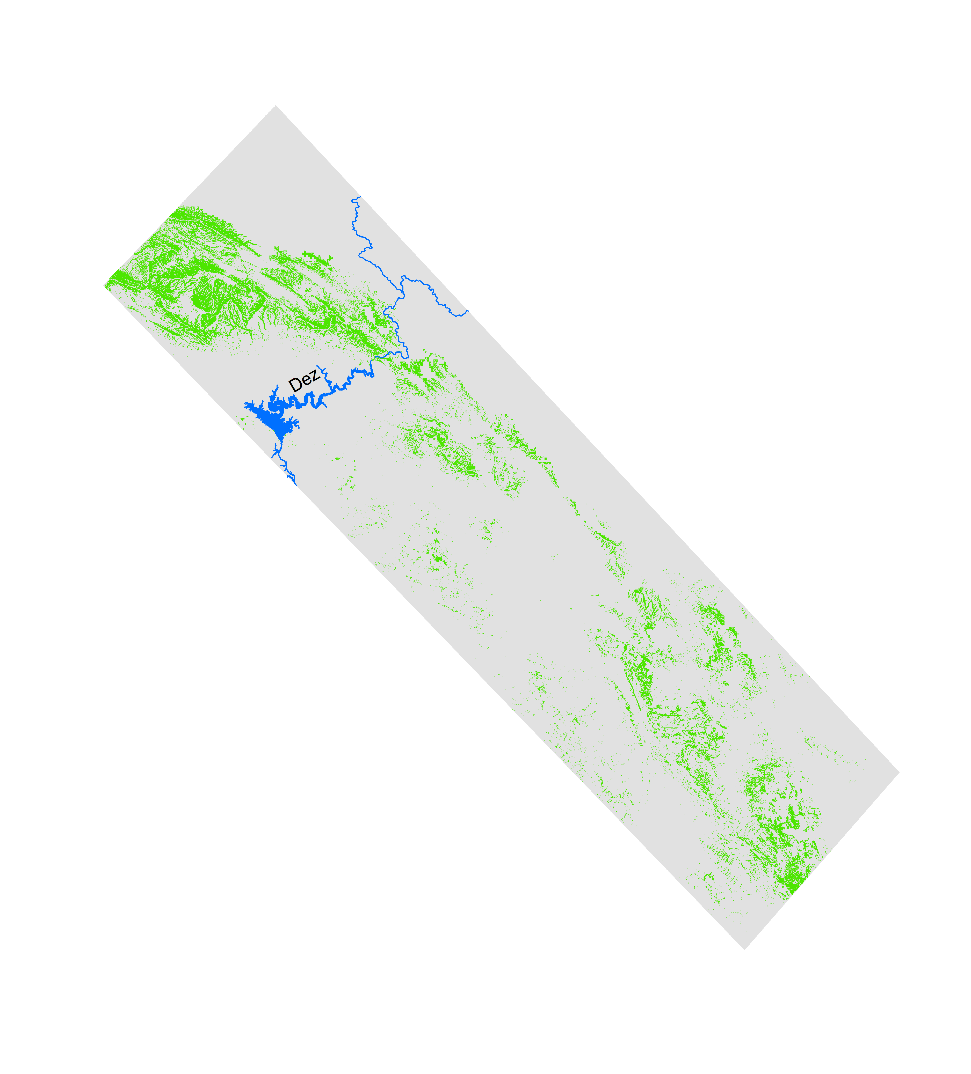

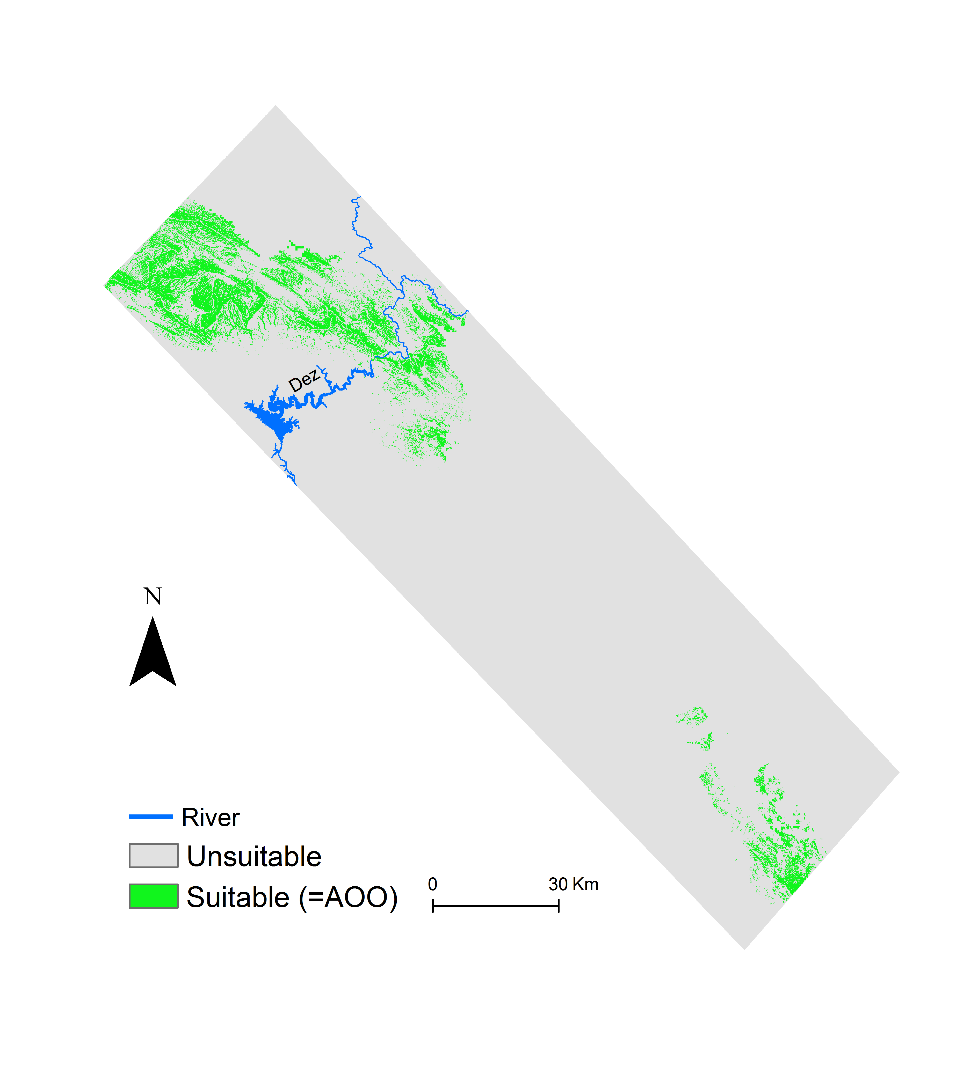


(a)

(b)

**Supplementary Figure S4** Potential area of occupancy (pAOO) of *Neurergus* *kaiseri* as predicted by (a) Species-level model and (b) combined lineage-level model after eliminating nonspring areas.

**Supplementary Figure S5** The total estimate of the potential area of occupancy (pAOO) for *Neurergus kaiseri* after eliminating non-spring areas according to different spatial resolution (based on fine-scale habitat suitability and IUCN standard grain size) using species and within-species evolutionary levels.

Species locations

Landscape/ local variables

N/S Lineage locations

Species-level DM

Species-level landscape-DM

Species-level local-DM

Lineage–level landscape-DMs

Lineage-level

local-DMs

Maxent

Maxent

Overlaid lineages binary DMs

Combined lineage-level landscape-DM

Combined lineage-level local-DM

Overlaid landscape & local DMs

Lineage-level DM

Overlaid landscape & local DMs

Input Data

Model Output

Intermediate Stage

**Legend**

**Supplementary Figure S6** Steps in the distribution model (DM) at species and evolutionary lineage levels at the landscape and local spatial scales. The modelling process may be done using different presence datasets: whole localities for the species, and the presences which recorded separately for each evolutionary lineages/clades. Landscape and local distribution models were performed for each presence dataset in Maxent and then overlaid to finally produce species-level DM or lineage-level DM.

**Supplementary Note**

*Comparison of MTP and MTSS threshold selection methods*

Using a number of simulated species Liu, White et al. (2013) found that maximizing sensitivity and specificity (MTSS) is the most efficient method regarding omission errors for threshold selection. This result contrasts with our finding in using a “real” species of N. kaiseri demonstrated that MTSS resulted in both the greatest omission error (larger unsuitable areas) and increased loss of suitable areas than Minimum training presence (MTP).

**Supplementary Table S3** The number of pixels for each model and % of correct classification records for each of the lineages/species based on Maximum training specificity plus specificity (MTSS) and Minimum training presence (MTP) cut-off threshold

1. MTP/MTSS cut-off thresholds

| Cut-off Threshold |  | Landscape scale | | | Local scale | | |
| --- | --- | --- | --- | --- | --- | --- | --- |
|  |  | Species-Level | Lineage-Level | | Species-Level | Lineage-Level | |
|  |  |  | N | S |  | N | S |
| MTSS | N of suitable pixels (30m) | 1,841,056 | 1,386,953 | 1,986,274 | 1,609,736 | 1,054,502 | 970,951 |
|  | % of correct classification | 64% | 88% | 40% | 82% | 83% | 80% |
| MTP | N of suitable pixels (30m) | 3,141,807 | 1,881,323 | 6,557,992 | 2,995,407 | 2,703,309 | 1,227,324 |
|  | % of correct classification | 85% | 94% | 90% | 96% | 94% | 90% |

1. Percent of correct classification of each clade’s records by another model based on MTP and MTSS.

|  | MTP | | | | MTSS | | | |
| --- | --- | --- | --- | --- | --- | --- | --- | --- |
| Models  clade | Landscape scale | | Local scale | | Landscape scale | | Local scale | |
|  | N | S | N | S | N | S | N | S |
| N (18 records) | 94% | 0 | 94% | 55% | 88% | 0 | 83% | 90% |
| S (10 records) | 0 | 90% | 90% | 90% | 0 | 40% | 60% | 80% |

1. Percent of correct classification of lineage-level model in comparison with species-level model.

|  | MTP | |  | | MTSS | |
| --- | --- | --- | --- | --- | --- | --- |
| Taxonomic level | Landscape scale | Local scale |  | Landscape scale | | Local scale |
| Lineage level | 92% | 92% |  | | 71% | 82% |
| Species level | 85% | 96% |  | | 64% | 82% |

**References**

1 Drusch, M. *et al.* Sentinel-2: ESA's optical high-resolution mission for GMES operational services. *Remote. Sens. Environ* **120**, 25-36, (2012).

2 Ma, J., Lin, G., Chen, J. & Yang, L. in *2010 18th International Conference on Geoinformatics.* 1-4.

3 Pourtaghi, Z. S. & Pourghasemi, H. R. GIS-based groundwater spring potential assessment and mapping in the Birjand Township, southern Khorasan Province, Iran. *Hydrogeology Journal* **22**, 643-662, (2014).

4 Liu, C., White, M. & Newell, G. Selecting thresholds for the prediction of species occurrence with presence‐only data. *J. Biogeogr.* **40**, 778-789, (2013).
